# Unveiling the Effectiveness of Agency Cost and Firms’ Size as Moderators Between CSR Disclosure and Firms’ Growth

**DOI:** 10.3389/fpsyg.2020.01624

**Published:** 2020-08-13

**Authors:** Aswad Akram, Yingkai Tang, Jasim Tariq

**Affiliations:** ^1^Business School, Sichuan University, Chengdu, China; ^2^Department of Business Administration, Iqra University, Islamabad, Pakistan

**Keywords:** corporate social responsibility, agency cost, firm size, firm growth, Pakistani companies

## Abstract

The excellence of corporate governance in companies lies in their ability to adopt the corporate social responsibility (CSR), which enhances their growth. This study examines the effect of agency cost, firm size, and CSR disclosure on the firms’ growth. Specifically, the study analyzed the agency cost and firms’ size as the moderators that influence the firms’ performance asymmetrically. In its approach, the study compiled data of 300 Pakistani listed companies, which have a significant concern with CSR for the period 2010–2018. Using the 2SLS and GMM instrumental panel regressions, our empirical results show that the agency cost is detrimental to the firms’ growth. In contrast, the firms’ size boosts the firms’ growth. Moreover, the growth of firms with leverage declines and the presence of independent directors improves the firms’ growth.

## Introduction

The extant literature enunciates how corporate governance is significant for all companies (Daily et al., 2002; Gabrielsson, 2017). The corporate governance concept basically entails leading or guiding (Abdullah and Benedict, 2009). With corporate governance, all types of firms, including novel entrepreneurs to whom corporate governance is inevitable, can confront the challenging business environment. Many theories (agency, resource dependence, stewardship, transaction cost, stakeholder, and political theories) explore the efficiency of corporate governance and negative aspects that can be detrimental to the corporate governance. Stewardship, stakeholder, and resource-based theories enunciate the role of managers and executives when working with the stakeholders, thus enhancing the growth of the firm. The transaction cost and agency theories—detrimental to a firm’s growth—are, however, beneficial to the top executives. Political theory highlights the pros and cons of political links within and outside the organization. According to Zattoni, the research on corporate governance has been conducted under the umbrella of the agency theory, which considers the economic factor to highlight the effectiveness of the corporate governance (Zattoni et al., 2013). The theory of corporate governance also suggests that the interests of minority shareholders should be considered (by mitigating the agency cost; Chen et al., 2019).

Corporate governance acts as vanguard to the firms’ performance (Adams et al., 2010; Bhagat and Bolton, 2019). Since the inception of globalization, products have become an integral part of human life globally. However, societies are faced with changes in organizations and environmental pollution; thus, firms are recommended to adopt corporate social responsibility (CSR) measures to alleviate these concerns. Meanwhile, to confront the dynamic environment of business, organizations are oriented toward innovative activities. These innovative activities, however, should be aligned with the CSR. Some studies posit that high-performing firms always adopt CSR measures, whereas low-performing ones are less likely to adopt these measures. Large firms perform well generally. However, the firm size as a moderator of firm growth has not been explored comprehensively.

Owing to a dynamic business environment, many developed and developing countries are transitioning to industrialization, which eventually pollutes the environment. On the one hand, developed countries adopt CSR measures to alleviate the environmental problems. Environmental and corporate governance activities affect the performance of businesses (Xie et al., 2019). Large firms are oriented toward the CSR; they are also donating their funds to charities. Through this contribution, firms are working for the benefit of the society. Disclosing CSR measures boosts performance of firms (Akben-Selcuk, 2019). On the other hand, some developing countries orient their governmental institutions to adopt CSR measures^1^ in order to meet the international standards of organizational structures (Saeidi et al., 2015).

Being an emerging economy, Pakistan is suffering from severe economic decline (Javeed and Lefen, 2019). Moreover, Pakistani firms are confronting the problems of low-quality manufacturing products, inadequate infrastructure for living and lack of laborers’ law. Meanwhile, due to mismanagement of wastage material among Pakistani firms, there is continuous threat of pollution and environmental issue (Ehsan et al., 2018). In a Pakistani perspective, it has been witnessed that matured firms endorse CSR activity as compared to small firms (Waheed, 2005). Significantly, SECP (security exchange commission of Pakistan) had introduced the CSR disclosure measure in 2009, which is also quite novel (Javeed and Lefen, 2019). Moreover, the intervention of government among Pakistani SOEs^2^ is also doubtful due to their sluggish performance (Bhat et al., 2018). Henceforth, in such circumstances, it is quite interesting to analyze the effectiveness of CSR on Pakistani firms under the influence of the specific moderators.

Comprehensively, CSR activity agitates the problem caused by spending extra funds by the upper echelon (McGuinness et al., 2017). Thus, it would also be worthwhile to contemplate the impact of the CSR disclosure on firms’ performance under the moderating influence of firm size. More specifically, it is quite significant to analyze the effectiveness of agency cost as a moderator between CSR disclosure and firms’ growth. Arguably, being an emerging country, Pakistani firms have been suffering from agency cost problem, which is why this study will contemplate whether the agency cost problem exacerbates the firms’ performance or not under the adaptation of CSR measures.

The remnant of the paper proceeds as follows. *Section “Literature review and hypothesis formulation”* describes the literature review and hypotheses formulation. *Section “Data and variable measures”* illustrates the data accumulation and variable measures along with empirical models. *Section “Empirical results”* illustrates the empirical results. *Section “Empirical Results of GMM instrumental Regression”* signifies the results of GMM instrumental regression. *Section “Discussion and Conclusion”* elucidates the conclusion and practical implications.

## Literature Review and Hypothesis Formulation

Argumentatively, it is the prime responsibility of the organization to take care of society and actively participate in such activities that are beneficial for the society. In this regard, legitimate theory emphasizes on the aspect that an organization should disclose its information about CSR to the public and also execute such strategies that are conducive for environment. Further, in the context of social reporting, the legitimacy theory seems to be widely applicable (Fernando and Lawrence, 2014). Meanwhile, some other aspect of the legitimacy theory emphasizes on the dynamic phenomenon that divulges the corporate objectives under the influence of public desire while confronting the societal expectations (Deegan et al., 2002). Hence, it is essential for the firms to be responsible for securing its surroundings, which can be achieved via adopting CSR. Adopting CSR can be costly, and its advantage may not be apparent; hence, stakeholders are not entirely satisfied with the measures (Gul et al., 2020). Some studies showed how CSR disclosure is measured. According to Gatti and Seele, European reports are more focused on the employees’ issues, while American reports are not (Gatti and Seele, 2014). Although different terminologies are coined for CSR, there is no clarification what it really means. Among others, these terminologies include “sustainable development,” “corporate citizenship,” “sustainable entrepreneurship,” “Triple Bottom Line,” “business ethics,” and CSR. Sharif and Rashid suggested that corporate governance influences the CSR (Sharif and Rashid, 2014). They have examined the role of non-executive directors and found that they influence the CSR reporting of banks positively. Furthermore, they enunciated that corporate governance plays a pivotal role in boosting the CSR disclosure activity. They have encapsulated that CSR activity is the indicator of the firms’ economic conditions (firms’ market position, size, industry relationship, risk management, market response, micro and macro environmental impact, and companies’ good will). Even more, they clarified that upper echelon work for the beneficiary of stakeholders.

Other studies suggest that CEO ownership negatively influences the CSR activities (Elgergeni et al., 2018). They have formulated voluntary CSR by considering its different aspects. Gender difference has also been analyzed to demonstrate its effect on CSR. They have mentioned that CSR activity is conducive for the company’s internal and external mechanisms. More precisely, CSR in companies not only enhances the revenue through its good reputation, but also mitigates the risk through nourishing the relation with customers. The studies further suggested that women are more oriented toward ethical issues as compared to men (Simionescu, 2015). A diverse board reassures stakeholders that the organization is capable to deal with social responsibilities. Moreover, an external director ownership is linked to improved CSR activities (Oh et al., 2019).

Corporate social responsibility can be regarded as a vehicle for enhancing the firms’ performance. In this regard, many studies have witnessed that CSR affects the performance asymmetrically (Broadstock et al., 2019). Meanwhile, it should be emphasized that CSR is also beneficial for firms’ growth.

However, the effect of CSR on firms’ performance is still subject to an in-depth study that considers the agency cost and firms’ size. This leads us to the first hypothesis.

H1: CSR disclosure positively influences the firm performance.

Existing studies already indicated that CSR enhances the firms’ performance (Su and Sauerwald, 2018), suggesting a positive relationship between CSR and firms’ growth. Moreover, the studies suggested that firms’ size has a positive relation with the firms’ growth (Shaukat et al., 2016). Meanwhile, it has also been demonstrated that board independence and gender diversity are necessary for escalating the growth and CSR activity. Further, scholars have witnessed that corporate governance plays the role of a moderator when improving the CSR activities (Su and Sauerwald, 2018). In a recent study, Tulung and Ramdani (2018) have examined the positive relation between firms’ size and performance of a board. Other studies suggest that firm’s size boost innovative performance of the firms (Jugend et al., 2018). Similarly, Yook et al. (2018) have demonstrated the firms’ size as moderator while influencing the environmental issue but neglected CSR disclosure and CSR performance which can affect the firms’ performance. The firms’ size plays a significant role in boosting the firms’ growth. It also boosts the CSR performance by ameliorating the sustainability reporting (Schreck and Raithel, 2018). Therefore, it can be conjectured that the firms’ size can act as a moderator between the CSR and performance while boosting the firms’ growth.

H2: Firm size as a moderator positively influences the firms’ performance via CSR activity.

Intuitively, pragmatic legitimacy theory enunciates that it is the responsibility of the firms to disclose the CSR. Meanwhile influential legitimacy emphasizes on the interests of stakeholders (Clarkson, 1995). Hence, it can be encapsulated that influential legitimacy theory suggests to alleviate the agency problem while providing benefits to the stakeholders. Categorically, the effective corporate mechanism always endeavors to mitigate the principal agent problem. Agency cost problem always emerges whenever the target of principal and agent is not congruent. The extant literature has witnessed that agency cost problem is reduced by adopting CSR activities (Li et al., 2017), which will eventually improve performance. In a recent study, it has been demonstrated that CSR disclosure and agency cost are not related (Zhou et al., 2018). Meanwhile the firms’ performance has a positive impact on the CSR (Erhemjamts et al., 2013), while agency cost mitigates the firms’ growth (Anderson et al., 2018). Comprehensively, CSR activity agitates the problem that is caused by spending extra funds by the upper echelon (McGuinness et al., 2017). Thus, it would also be worthwhile to contemplate the impact of the CSR activity on firms’ performance under the influence of agency cost problem. The extent literature has witnessed that agency cost problem is reduced by adopting CSR activities (Li et al., 2020), which ultimately escalates the performance. In a recent study, it has been demonstrated that CSR disclosure and agency cost has a negative relation (Chen et al., 2019). Meanwhile the firms’ performance has a positive impact on the CSR. Hence, it can be assessed that the result should be negative whether agency cost acts as a moderator between performance and CSR. In this regard, we can formulate our hypothesis.

H3: Agency cost as a moderator will mitigate the performance in the presence of CSR activity.

**FIGURE 1:**
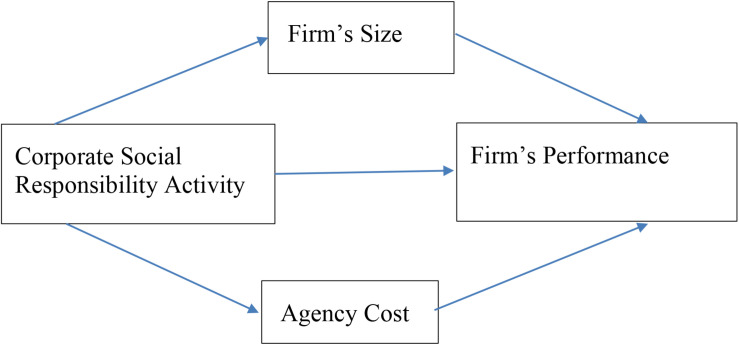
The theoretical model of research.

On the basis of previous discussion, this study intends to explore the CSR role on firm performance with the moderating effects of firm size and agency cost as shown in Figure 1.

## Data and Variable Measures

We have collected data of the firms listed on the Karachi Stock Exchange (KSE) for the period 2010–2018, Among 553 firms listed on the KSE, 300 selected firms have been witnessed to be highly concerned with CSR activity. The mathematical expression below indicates CSR disclosure. See specific attributes in section “Appendix”.

(1) $${\text{CSRD}}_{i,t}=\sum {\text{X}}_{\text{it}}\,/\,\text{n}\,\text{where}\,\text{n}\in {\text{Z}}^{+}$$

Equation (1) illustrates CSR disclosure as the sum of all attributes. Further, in this equation, “X_it_” indicates the total number of attributes.

For empirical analysis, we have endorsed the control variables and independent variables such as “LnTA” (total assets), “EPS” (earnings per share), “Leverage,” “SOE” (state-owned enterprises), and number of independent directors (Chen et al., 2019; Sarfraz et al., 2019; Shah et al., 2019b). “EPS” and “lnTA” capture the firms’ performance (Li et al., 2020; Sarfraz et al., 2020). Additionally, “leverage” signifies the economic condition of the firm, whereas independent directors always monitor sustainability of the firm’s growth. Meanwhile, “ROA” and “ROE” have been endorsed as proxies for measuring the firm performance.

### Empirical Models

To demonstrate the impact of CSR on firms’ growth via agency cost and firm size, we used the panel regression technique. The threat of endogeneity has been eradicated through 2SLS instrumental regression. Some scholars suggest interpreting the results of 2SLS instrumental regression directly (Larcker and Rusticus, 2010; Shah et al., 2019a; Sarfraz et al., forthcoming). Therefore, we show only the results of 2SLS regression. “Normative CSR disclosure” has been endorsed as an instrumental variable. The empirical models are expressed as follows:

$$F\,P_{i\,t}=\gamma_{0}+\gamma_{1\,i\,t}\,\left(C\,S\,R\,D_{i\,t}^{*}\,A\,g\,e\,n\,c\,y\,C\,o\,s\,t_{i\,t}\right)+\gamma_{2\,i\,t}\,S\,O\,E_{i\,t}$$

$$+\;{\text{γ}}_{3it}INDDIR_{it}+{\text{γ}}_{4it}FS_{it}+{\text{γ}}_{5it}lnTA_{it}$$

$$+\gamma_{6\,i\,t}\,L\,e\,v\,e\,r\,a\,g\,e_{i\,t}+\gamma_{7\,i\,t}\,E\,P\,S_{i\,t}+\tau \,I\,n\,d\,u\,s\,t\,r\,y$$

(2) $$d\,u\,m\,m\,y+\mu \,Y\,e\,a\,r\,d\,u\,m\,m\,y+\varepsilon_{i\,t}$$

$$F\,P_{i\,t}=\gamma_{0}+\gamma_{1\,i\,t}\,\left(C\,S\,R\,R_{i\,t}^{*}\,F\,S_{i\,t}\right)+\gamma_{2\,i\,t}\,S\,O\,E_{i\,t}+\gamma_{3\,i\,t}$$

$$I\,N\,D\,D\,I\,R_{i\,t}+\gamma_{4\,i\,t}\,l\,n\,T\,A_{i\,t}+\gamma_{5\,i\,t}\,L\,e\,v\,e\,r\,a\,g\,e_{i\,t}$$

$$+\gamma_{6\,i\,t}\,E\,P\,S_{i\,t}+\tau \,I\,n\,d\,u\,s\,t\,r\,y$$

(3) $$d\,u\,m\,m\,y+\mu \,Y\,e\,a\,r\,d\,u\,m\,m\,y+\varepsilon_{i\,t}$$

$$F\,P_{i\,t}=\gamma_{0}+\gamma_{1\,i\,t}\,C\,S\,R\,D_{i\,t}+\gamma_{2\,i\,t}\,S\,O\,E_{i\,t}+\gamma_{3\,i\,t}\,I\,N\,D\,D\,I\,R_{i\,t}$$

$$+\gamma_{4\,i\,t}\,F\,S_{i\,t}+\gamma_{5\,i\,t}\,l\,n\,T\,A_{i\,t}+\gamma_{6\,i\,t}$$

$$L\,e\,v\,e\,r\,a\,g\,e_{i\,t}+\gamma_{7\,i\,t}\,E\,P\,S_{i\,t}+\tau \,I\,n\,d\,u\,s\,t\,r\,y$$

(4) $$d\,u\,m\,m\,y+\mu \,Y\,e\,a\,r\,d\,u\,m\,m\,y+\varepsilon_{i\,t}$$

In Eqs (2, 3), the dependent variable is firms’ performance, indicated by “FP_it_.” The variables “FS_it_” and “INDDIR_it_” are firm size and number of independent directors, respectively. Meanwhile, the moderators have been represented by the interaction terms “CSRD_it_*AgencyCost_it_” and “CSRD_it_*FS_it_,” respectively. Moreover, Eq. (4) represents the panel regression for analyzing the effectiveness of CSR disclosure on firms’ performance. The variables “τ*Industrydummy* + μ*Yeardummy*” represents the industry dummy and year dummy.

## Empirical Results

Firstly, fixed effect panel regression has been confirmed with the Hausman test. Additionally, lagged variable regression has been executed while confirming the presence of an endogeneity problem. However, we have represented the authentic 2SLS instrumental regression results. Table 1 illustrates the descriptive statistics. All the variables but the agency cost (due to some missing data, the number of observations is 2286) were equally observed. Agency cost has been measured through the proxy asset utilization ratio. The asset utilization ratio is determined as annual sales/total assets.

**TABLE 1:** Descriptive statistics.

**Variables**	**Obs**	**Mean**	**Std. Dev**	**Min**	**Max**
CSRD	2400	0.6140385	0.1195372	0.2307692	0.9230769
EPS	2396	0.3372983	1.13133	−3.859921	42.43205
ROA	2399	0.298656	0.2483674	−2.776046	8.441391
ROE	2366	0.2717825	0.5928742	−0.645726	9.392855
LNTA	2397	22.29809	1.477981	15.97917	30.60502
LNEMP	2398	7.793752	1.420357	1.609438	13.12851
Agency cost	2286	6.539559	5.15853	0	350.1522
Leverage	2399	0.4916175	0.3290587	0.007969	8.611787
SOE	2400	0.4504167	0.4976391	0	1
INDDIR	2400	7.960417	0.995121	4	13

Table 1 illustrates the descriptive statistics. Agency cost has the maximum standard deviation. The minimum value of the agency cost is “0” and maximum value is 350.1522, with a mean value of 6.539559. Thus, its volatility is high compared to remaining variables, but it is acceptable for empirical analysis.

Table 2 indicates the correlation matrix, which reveals that regression can be run without any threat of multicollinearity. The maximum correlation value is “0.3594” between LNEMP and LNTA.

**TABLE 2:** Correlation matrix.

**Variables**	**CSRD**	**EPS**	**ROA**	**LNTA**	**LNEMP**	**SOE**	**Agency cost**	**INDDIR**	**Leverage**	**ROE**
CSRD	1.000									
EPS	–0.0055	1.000								
ROA	–0.0027	0.2181	1.000							
LNTA	–0.0292	0.1497	–0.0187	1.000						
LNEMP	0.0561	0.0727	0.0005	0.3594	1.000					
SOE	–0.1079	0.0014	–0.0208	0.1646	0.1104	1.000				
Agency cost	0.0157	0.0080	0.0110	–0.0452	–0.0603	–0.0449	1.000			
INDDIR	0.0094	0.0129	–0.0218	0.2898	0.3551	0.1075	–0.0407	1.000		
Leverage	–0.0571	–0.1064	–0.3566	0.1757	0.0539	0.1023	–0.0446	0.0719	1.000	
ROE	0.0162	–0.0103	0.0142	–0.0214	–0.0239	0.0038	0.0253	–0.0304	–0.0034	1.000

In Table 2, the maximum correlation value “0.3594” is acceptable for empirical analysis. All other variables have less correlation values, which shows that there is no threat of absolute multicollinearity among all variables.

Table 3 shows 2SLS regression results. CSR disclosure has enhanced the firms’ growth (first row of Table 3). Conversely, firm size has reduced the firms’ growth (fifth row of Table 3), because a large number of employees can impede the firms’ growth. Meanwhile, the numbers of independent directors have boosted the CSR activity, because the external directors can compel the top executives to disclose the CSR, thus improving the firms’ performance.

**TABLE 3:** 2SLS Instrumental regression for CSR disclosure and firms’ performance.

**Variables**	**(1)**	**(2)**	**(3)**	**(4)**	**(5)**
	**ROA**	**ROA**	**ROA**	**ROE**	**ROE**
CSRD	2.269*	2.269*	2.260*	3.830*	3.838*
	(1.438)	(1.433)	(1.438)	(1.833)	(1.832)
EPS	–0.00124	–0.00115	–0.00141	–0.00231	–0.00216
	(0.0124)	(0.0124)	(0.0124)	(0.0162)	(0.0162)
Leverage	0.0381	0.0370	0.0379	0.0261	0.0263
	(0.0477)	(0.0480)	(0.0477)	(0.0508)	(0.0508)
LNTA	–0.000597	–0.00222	–0.000521	–0.00175	–0.00182
	(0.0107)	(0.0108)	(0.0107)	(0.0114)	(0.0114)
LNEMP	−0.0298**	−0.0301**	−0.0299**	−0.0418**	−0.0417**
	(0.0151)	(0.0152)	(0.0151)	(0.0161)	(0.0161)
SOE	0.0658	0.0696	0.0651	0.1769	0.1775
	(0.0426)	(0.0435)	(0.0426)	(0.0454)	(0.0454)
INDDIR	0.0966*	0.107*			0.0842*
	(0.0110)	(0.0114)			(0.0117)
Industry _Dummy_	YES	YES	YES	YES	YES
Year _Dummy_	YES	YES	YES	YES	YES
Constant	–1.146	–1.109	–1.104	–1.923	–1.960
	(0.903)	(0.895)	(0.907)	(0.967)	(0.962)
Observations	2,357	2,308	2,357	2,357	2,357
R-squared	0.389	0.376	0.389	0.276	0.276

Standard errors in parentheses *** *p* < 0.01, ** *p* < 0.05, and * *p* < 0.1.

Table 3 indicates that CSR disclosure is positively significant for ROA and ROE. The coefficient values of CSRD are “2.269^∗^” and “3.830^∗^,” respectively. Further, the variable “LNEMP” is negatively significant for ROA and ROE (“−0.0298^∗∗^” and “−0.0418^∗∗^,” respectively). Reciprocally, the variable “INDDIR” is positively significant for both ROA and ROE (“0.0966^∗^” and “0.0842^∗^,” respectively).

Table 4 shows that firms with a large number of employees can boost growth by disclosing the CSR. Moreover, the role of independent directors is highly appreciable through their vigilant strategies (as NIND is positively significant).

**TABLE 4:** 2SLS Instrumental regression for moderator firms’ size.

**Variables**	**(1)**	**(2)**	**(3)**	**(4)**
	**ROA**	**ROA**	**ROE**	**ROE**
CSRD*FS	0.353*	0.352*	0.489*	0.489*
	(0.201)	(0.201)	(0.275)	(0.275)
EPS	–0.00106	–0.00121	–0.00197	–0.00209
	(0.0126)	(0.0126)	(0.0135)	(0.0135)
Leverage	0.0364	0.0362	0.0244	0.0242
	(0.0482)	(0.0482)	(0.0514)	(0.0514)
LNTA	–0.00370	–0.00362	–0.00524	–0.00517
	(0.0107)	(0.0106)	(0.0114)	(0.0114)
SOE	0.0644	0.0638	0.0760	0.0755
	(0.0428)	(0.0429)	(0.0477)	(0.0478)
INDDIR	0.0951*		0.0715*	
	(0.0113)		(0.0101)	
Industry _Dummy_	YES	YES	YES	YES
Year _Dummy_	YES	YES	YES	YES
Constant	0.462**	0.494**	0.516**	0.543**
	(0.224)	(0.219)	(0.239)	(0.234)
Observations	2,357	2,357	2,357	2,357
R-squared	0.386	0.386	0.273	0.273

Standard errors in parentheses *** *p* < 0.01, ** *p* < 0.05, and * *p* < 0.1.

Table 4 indicates that the interaction term of CSRD and firms’ size (CSRD^∗^FS) is positively significant for both ROA and ROE (“0.353^∗^” and “0.489^∗^,” respectively). Meanwhile, INDDIR is also positively significant for firms’ growth (“0.0951^∗^” and “0.0715^∗^,” respectively).

Table 5 shows the role of the agency cost as the moderator. It shows that the agency cost reduces the firms’ growth (first row of Table 5), because it disregards CSR disclosure and performance. As a result, it affects the firms’ growth and image adversely. Furthermore, leverage shows negative significance, which also threatens the firms’ performance, because investors are easily discouraged by existing heavy loans.

**TABLE 5:** 2SLS Instrumental regression for the moderator agency cost.

**Variables**	**(1)**	**(2)**	**(3)**	**(4)**	**(5)**
	**ROA**	**ROA**	**ROA**	**ROE**	**ROE**
CSRD*AGC	−0.0567***	−0.0584***	−0.0581**	−0.0645**	−0.0642**
	(0.0431)	(0.0391)	(0.0390)	(0.0421)	(0.0420)
EPS	0.00145	0.00151	0.00140	0.000861	0.000766
	(0.0158)	(0.0154)	(0.0154)	(0.0166)	(0.0165)
Leverage	−0.424***	−0.0463***	−0.0460***	−0.0569*	−0.0566*
	(0.0615)	(0.0603)	(0.0601)	(0.0699)	(0.0698)
LNTA		–0.0219	–0.0218	–0.0350	–0.0349
		(0.0151)	(0.0150)	(0.0162)	(0.0162)
SOE		–0.0331	–0.0331	–0.0421	–0.0421
		(0.0447)	(0.0446)	(0.0481)	(0.0480)
INDDIR	0.00575	0.00564		0.00481	
	(0.00753)	(0.0136)		(0.0147)	
Industry _Dummy_	YES	YES	YES	YES	YES
Industry _Dummy_	YES	YES	YES	YES	YES
Constant	−0.815*	0.861**	0.879**	0.956**	0.971**
	(0.389)	(0.387)	(0.386)	(0.417)	(0.415)
Observations	2,245	2,245	2,245	2,245	2,245
R-squared	0.326	0.347	0.326	0.258	0.258

Standard errors in parentheses *** *p* < 0.01, ** *p* < 0.05, and * *p* < 0.1.

In Table 5, the moderator agency cost has negatively affected ROA and ROE. The first row of Table 5 indicates that the coefficient values for the interaction term (CSRD^∗^AGC) are “−0.0567^∗∗∗^” and “−0.0645^∗∗^,” respectively. Similarly, the variable “leverage” is also negatively significant for firms’ performance. The coefficient values of the variable “leverage” are “−0.424^∗∗∗^” and “−0.0569^∗^” (third row of Table 5), respectively. The remaining variables LNTA (logarithm of total assets), EPS (earnings per share), SOE (state owned enterprises), and INDDIR (independent directors) are insignificant.

## Empirical Results of GMM Instrumental Regression

In this section, we have executed the GMM instrumental regression. The objective of executing the GMM instrumental regression is to confirm that our results are authenticated and reliable to be interpreted for implications. Table 6 evaluates the impact of CSR disclosure, which positively boosts the performance. Table 6 elaborates that results are the same (Table 3). Additionally, the variable “LNEMP” has indicated the negative significance.

**TABLE 6:** GMM for Corporate social responsibility disclosure and firm’s performance.

**Variables**	**(1)**	**(2)**	**(3)**	**(4)**
	**ROA**	**ROA**	**ROE**	**ROE**
CSRD	2.125*	2.116*	2.398*	2.560*
	(1.502)	(1.498)	(1.374)	(1.413)
EPS	0.0418	0.0418	–0.00192	–0.00141
	(0.0295)	(0.0295)	(0.00971)	(0.0100)
Leverage	–0.224	–0.224	0.0365	0.0379
	(0.156)	(0.156)	(0.0368)	(0.0377)
LNTA	0.00645	0.00624	0.000844	–0.000521
	(0.0130)	(0.0131)	(0.0103)	(0.0102)
LNEMP	−0.0251*	−0.0253*	−0.0258*	−0.0299**
	(0.0102)	(0.0102)	(0.0138)	(0.0139)
SOE	0.0552	0.0549	0.0630*	0.0651*
	(0.0620)	(0.0618)	(0.0374)	(0.0383)
INDDIR	–0.000809		–0.00737	
	(0.00316)		(0.00706)	
Industry _Dummy_	YES	YES	YES	YES
Year _Dummy_	YES	YES	YES	YES
Constant	–1.224	–1.218	–0.999	–1.104
	(1.593)	(1.592)	(0.872)	(0.898)
Observations	2,380	2,380	2,357	2,357
R-squared	0.298	0.298	0.210	0.210

Standard errors in parentheses *** *p* < 0.01, ** *p* < 0.05, and * *p* < 0.1.

Table 6 indicates that CSRD is positively significant for performance. Significantly, the first row of Table 6 indicates the coefficient values of CSRD (2.125^∗^ and 2.398^∗^, respectively). Additionally, the variable “LNEMP” is negatively significant, whose coefficients values are “−0.0251^∗^” and “−0.0299^∗∗^,” respectively. However, the remaining variables have shown insignificant results.

Table 7 has shown that the firms’ size as a moderator has positively boosted the firms’ performance (as indicated by the first row). Moreover, the leverage has shown negative significance.

**TABLE 7:** GMM for firms’ size as a moderator.

**Variables**	**(1)**	**(2)**	**(3)**	**(4)**
	**ROA**	**ROA**	**ROE**	**ROE**
CSR*FSZ	0.320**	2.376**	0.907*	3.889*
	(0.187)	(1.36)	(0.397)	(2.56)
EPS	0.0444	0.0213	–0.0157	0.0484
	(0.0312)	(0.141)	(0.0244)	(0.343)
LEV	−0.294*	–0.00731	−0.0943*	−0.571*
	(0.171)	(1.694)	(0.015)	(0.380)
LNTA	0.0552	–0.506	–0.138	1.230
	(0.0646)	(3.497)	(0.132)	(7.270)
SOE	–0.0221	0.139	0.0792	–0.305
	(0.0343)	(0.922)	(0.0842)	(1.840)
INDDIR	0.0423		–0.115	
	(0.0518)		(0.106)	
Industry _Dummy_	YES	YES	YES	YES
Year _Dummy_	YES	YES	YES	YES
Constant	0.0955	–0.162	0.415	1.540
	(0.203)	(1.974)	(0.382)	(6.812)
Observations	2,391	2,391	2,357	2,357
R-squared	0.263	0.263	0.196	0.196

Robust standard errors in parentheses *** *p* < 0.01, ** *p* < 0.05, and * *p* < 0.1.

Table 7 has indicated that the moderator (firms’ size) positively boosts the performance. The first row of Table 7 indicates the coefficient values of the interaction term (CSR^∗^FSZ), which are “0.320^∗∗^” and “3.889^∗^,” respectively, Additionally, leverage has shown negative significance, whose coefficient values are “−0.294^∗^” and “−0.571^∗^,” respectively. Meanwhile, the remnant variables “EPS,” “SOE,” “INDDIR,” and “LNTA” are insignificant.

Table 8 indicates that agency cost is negatively significant for the firms’ performance. Suggestively, the results indicate that agency cost is even detrimental for firms’ performance whether a firm discloses the CSR. Moreover, firms having high foreign loan burden is detrimental for the firms’ growth. Reasonably, a firm that is suffering from loan burden cannot pay attention toward CSR activity. Meanwhile, agency cost exacerbates the already miserable plight that destroys the firms’ performance vehemently.

**TABLE 8:** GMM for agency cost as a moderator.

**Variables**	**(1)**	**(2)**	**(3)**	**(4)**
	
	**ROA**	**ROA**	**ROE**	**ROE**
CSR*AGC	−0.0608**	−0.0612**	−0.105*	−0.113*
	(*0.0148*)	(*0.0165*)	(*0.0069*)	(*0.0053*)
EPS	0.0415	0.0417	0.00525	0.00716
	(0.0327)	(0.0331)	(0.0129)	(0.0140)
LEV	−0.271*	−0.271*	–0.0807	–0.0896
	(0.144)	(0.144)	(0.0900)	(0.0958)
LNTA	−8.80*e*−05	–0.000598	–0.0147	–0.0208
	(0.0248)	(0.0268)	(0.0212)	(0.0238)
LNEMP	–0.00287	–0.00367	–0.0285	–0.0396
	(0.0183)	(0.0215)	(0.0315)	(0.0368)
SOE	–0.00345	–0.00410	–0.0561	–0.0657
	(0.0479)	(0.0505)	(0.0653)	(0.0703)
INDDIR	–0.00165		–0.0225	
	(0.00685)		(0.0165)	
Industry _Dummy_	YES	YES	YES	YES
Year _Dummy_	YES	YES	YES	YES
Constant	0.207	0.211	1.239	1.280
	(0.860)	(0.877)	(0.814)	(0.838)
Observations	2,277	2,277	2,243	2,243
R-squared	0.267	0.267	0.219	0.219

Robust standard errors in parentheses *** *p* < 0.01, ** *p* < 0.05, and * *p* < 0.1.

Table 8 has shown that agency cost as a moderator is negatively significant for firms’ performance (first row of Table 8). Additionally, leverage is negatively significant.

## Discussion and Conclusion

### Discussion

According to the corporate governance country assessment report launched by the World Bank in 2018, Pakistani firms are ameliorating its corporate governance structure. At the initial stage during 2001–2002, severe resistance among corporate organizations was observed, which later on accepted that corporate governance codes not only are significant but also proved to be conducive for these organizations. It has been observed that multinationals, renowned banks, and specifically family controlled firms have ameliorated their corporate structure via promulgating translucent corporate governance mechanism. In this regard, the current study signifies the role of corporate social responsible activity under the patronage of novel corporate governance, which influences the firms’ growth.

Empirical results have evaluated that CSR disclosure boosts the firms’ performance (Malik and Kanwal, 2018), suggesting that disclosing such activity not only boosts the performance but also allures the investors for being a philanthropist. Meanwhile, agency cost as a moderator has been signified as a detrimental vehicle for firms’ growth (Abdullah et al., 2012). Under the aegis of influential legitimacy theory, our results have been justified while suggesting to mitigate the agency cost problem; otherwise, CSR disclosure is useless. Moreover, the firms’ size as a moderator has boosted the firms’ performance (Tulung and Ramdani, 2018) while concluding that firms having a large number of employees have an extra opportunity to allocate their employees to concentrate on the corporate social responsible activities that is beneficial for the firms’ growth.

### Conclusion

Pakistan being an emerging economic and a vibrant member of CPEC, it is quite significant to contemplate Pakistani firms while confronting the devastated economy for the last 10 years. Pakistani firms have been compelled to adopt CSR activities, which makes it worthwhile to contemplate these firms due to the following reasons. Firstly, the Security Exchange Commission of Pakistan had promulgated the ordinance in 2009 to adopt CSR measures for the listed companies. Secondly, in 2017, the global climax index listed Pakistan as the seventh most vulnerable country confronting the climate change (Eckstein et al., 2016). Thirdly, being a member of CPEC countries, it would be interesting that Pakistan’s economy need to be escalated without disturbing the environment (Ikram et al., 2019). In this regard, the current study has elucidated the effectiveness of CSR disclosure on the firms’ performance under the aegis of agency cost and firms’ size.

We showed that CSR disclosure enhances the firms’ growth because investors are impressed by a firm’s involvement in corporate social responsibilities. Willingness to engage in CSR indicates the efficiency of the corporate governance. Furthermore, we found that the agency cost impedes the firms’ growth because it repels investors, who might end up diverting their investments elsewhere. Conversely, the firms’ size improves the firms’ growth because a firm can easily allocate its many employees to R&D and CSR activities, thus improving the performance. Generally, large firms are mature, with specific goals for growth. Such firms are more likely to adopt CSR activities, which will ultimately boost their growth. We also found that high leverage reduces the firms’ growth and independent directors boost the firms’ growth.

Through contemplation of this study, certain recommendations have been deduced for academicians, organizational scholars, and practitioners. Suggestively, the trend of CSR is conducive for the prosperity of the organizations, but specific steps are required to invigorate the intensity of this prosperity. Convincingly, firms should curtail the agency cost problem decisively, which not only tarnishes the images of the firms but also destroys its performance vehemently. Agency cost problem indicates that firms’ corporate governance mechanism is fragile, which ultimately allows the upper echelon to confiscate the rights of minority shareholders. Meanwhile, due to agency cost problem, firms spoil the funds, which is why the research and development department is neglected. Therefore, the firms’ prime priority is to eradicate the agency cost problem (either type 1 or type 2).

The study has practical implications for practitioners and academic scholars. First, the study recommends mitigating the agency cost problem because it may render CSR disclosure worthless. The agency cost impedes the firms’ growth. Secondly, the study suggests that firms should not worry about the firms’ size because it boosts the firms’ growth. Instead, firms should strive to mitigate the heavy loans as they affect growth adversely.

## Limitations of the Study

Our study has some limitations, which should be addressed by future research. First, the impact of CSR disclosure and not CSR performance has been analyzed on firms’ growth; therefore, future studies should focus on CSR performance as an excellent measure of growth. Second, the study considered only the agency cost as the moderator for growth, ignoring the immense effect of innovation as a significant tool for the firms’ growth. Thus, future research should consider innovation. Last, we have analyzed the impact of agency cost and firms’ size as moderators for Pakistan companies. Future studies can evaluate such effectiveness of agency cost and innovation as moderator for China and United States.

## Data Availability Statement

The raw data supporting the conclusions of this article will be made available by the authors, without undue reservation.

## Author Contributions

All authors listed have made a substantial, direct and intellectual contribution to the work, and approved it for publication.

## Conflict of Interest

The authors declare that the research was conducted in the absence of any commercial or financial relationships that could be construed as a potential conflict of interest.

## Footnotes

1 According to Saeidi et al. (2015), although CSR influences the firms’ performance directly, this direct effect is based on some indirect influence of other factors such as competitive advantage, reputation, and customer satisfaction.

2 Pakistani Airlines is struggling due to poor governance (Bhat et al., 2018).

## Appendix

### Detail of CSR Disclosure Activities

Companies in Pakistan are doing a lot of CSR activities. We have collected the information; list of activities is very much long. By keeping in view, the nature of activities related to CSR Disclosure, these activities are divided into the 7 main categories.

• Education.
• Community Development.
• Disaster Relief.
• Environment Plantation and Forestation.
• Healthcare.
• Water Provision and Purification.
• Infrastructure Development.
